# Integrating Single-Cell and RNA Sequencing to Predict Glioma Prognosis Through Lactylation

**DOI:** 10.3390/ijms27041649

**Published:** 2026-02-08

**Authors:** Ruyi Shen, Yinan Chen, Yan Li, Zhijie Lin

**Affiliations:** 1 The First Clinical Medical College, Faculty of Medicine, Yangzhou University, Yangzhou 225009, China; 222703116@stu.yzu.edu.cn (R.S.); 222201204@stu.yzu.edu.cn (Y.C.); 2 School of Traditional Chinese Medicine, Faculty of Medicine, Yangzhou University, Yangzhou 225009, China; 3 Key Laboratory of the Jiangsu Higher Education Institutions for Integrated Traditional Chinese and Western Medicine in Senile Diseases Control, Yangzhou University, Yangzhou 225009, China; 4 Department of Immunology, Faculty of Medicine, Yangzhou University, Yangzhou 225009, China

**Keywords:** glioma, lactylation, prognostic model, therapy

## Abstract

Gliomas are the most prevalent primary malignant neoplasms of the central nervous system, distinguished by their high recurrence rates and poor prognosis. Aerobic glycolysis in tumors generates excess lactate, which promotes lactylation, a post-translational modification (PTM). Although accumulating evidence implicates lactylation in glioma initiation and progression, previous lactylation-focused prognostic studies lacked single-cell resolution and broad validation, limiting their generalizability and clinical relevance. Single-cell and bulk RNA sequencing (RNA-seq) data were integrated to identify lactylation-enriched tumor cell populations and derive candidate genes. A risk model was developed using univariate Cox regression and the Least Absolute Shrinkage and Selection Operator (LASSO), and its predictive performance was validated in independent cohorts from the China Glioma Genome Atlas (CGGA). To improve clinical applicability, a nomogram integrating the risk score incorporating key clinical variables was constructed and externally validated. The risk groups showed distinct immune microenvironment profiles and differential drug sensitivity patterns. In this study, we established and validated a lactylation-related gene signature, with the derived risk score serving as a reliable prognostic biomarker for glioma. Furthermore, the model not only predicts overall survival (OS) but also exhibits the potential to inform drug selection and stratify patients for more precise and personalized therapeutic interventions.

## 1. Introduction

Gliomas are the most common primary malignant tumors of the central nervous system and are among the most aggressive. Their diffuse, infiltrative growth typically limits surgery to maximal safe resection rather than complete removal. Moreover, the blood–brain barrier restricts delivery of many therapeutic agents, contributing to frequent recurrence and persistently poor outcomes [1,2].

Glioblastoma (GBM), the most malignant subtype, progresses rapidly, recurs frequently, and exhibits intrinsic resistance to standard therapies. Despite advances in surgery, radiotherapy, and chemotherapy, these interventions rarely overcome the biological barriers posed by GBM. Consequently, GBM remains exceptionally lethal, and substantial improvements in overall survival (OS) have been limited [3].

Lower-grade gliomas (LGGs) grow more slowly and are generally associated with better outcomes, but they are not benign. They frequently recur and can progress to higher-grade, more aggressive gliomas over time [4]. Accordingly, management often aims to delay malignant progression. However, surgery can still cause substantial functional or cognitive deficits, compromising long-term quality of life [5].

These constraints underscore the need for reliable prognostic biomarkers and actionable therapeutic targets to enable more precise treatment and improved risk stratification.

Prognostic assessment still relies largely on histopathological grading and a limited set of molecular markers (e.g., isocitrate dehydrogenase (IDH) mutation, 1p/19q codeletion, and MGMT promoter methylation) [6,7]. Nevertheless, survival varies markedly even among patients with the same grade and molecular subtype [8]. This heterogeneity suggests that current markers do not fully capture glioma biology and motivates the development of biomarkers reflecting dynamic tumor states, including post-translational modifications (PTMs).

The Warburg effect—cancer cells’ preference for aerobic glycolysis—drives lactate accumulation that promotes angiogenesis, tumor growth, and immune evasion [9]. Lactate also supports lactylation, a PTM that can reshape gene expression and protein function [10]. Lactylation therefore links tumor metabolism to epigenetic regulation and may serve as both a biomarker and a therapeutic target. Dysregulation of lactylation-related genes has been associated with resistance to chemotherapy, immunotherapy, targeted therapy, and radiotherapy, implicating lactylation in treatment failure [11]. Accordingly, strategies that reduce lactylation may enhance therapeutic efficacy.

Recent studies have begun to investigate lactylation in glioma biology. For example, one study performed differential expression and clustering analyses of lactate metabolism-related genes and derived a 14-gene prognostic model strongly associated with the tumor immune microenvironment [12]. Another study constructed a lactylation-focused prognostic signature and experimentally validated a role for C19orf53 in promoting glioma cell migration and proliferation [13]. Together, these studies expand understanding of the glioma molecular landscape and support the search for new diagnostic markers and therapeutic strategies.

However, these analyses relied exclusively on bulk RNA sequencing (RNA-seq), limiting inference to the tissue level and precluding cell-resolved characterization. In addition, most studies were restricted to The Cancer Genome Atlas (TCGA): some pooled GBM and LGG, whereas others analyzed a single dataset, limiting robust risk stratification across subtypes. Finally, prior work largely focused on prognosis without clear clinical translation and rarely integrated drug-response data, further limiting clinical applicability.

Here, we aim to develop a prognostic model that is both clinically predictive and grounded in the biology of core tumor cell populations. We hypothesize that lactylation is heterogeneously distributed and preferentially enriched in tumor cell subpopulations that drive progression. Building on this hypothesis, we will develop a risk model that captures malignant potential by prioritizing cell-type-specific genes from these populations. The model is further designed to predict both prognosis and therapeutic responses, enabling stratified treatment across glioma grades and supporting individualized therapy selection.

## 2. Results

### 2.1. Study Workflow

As outlined in the workflow, the single-cell transcriptomic datasets GSE200984 and GSE141383 were first retrieved from the GEO database. Differential expression analysis was performed between the NPC subpopulation—characterized by markedly elevated lactylation within tumor tissues—and other cell types, and Venn analysis was used to identify the intersection of DEGs shared by the two cohorts. Then, transcriptomic profiles and clinical follow-up data of the TCGA glioma cohorts were extracted, the lactylation score of each sample was calculated by known lactylation-related genes, the samples were stratified into high- and low-score groups, and differential expression analysis was conducted to identify DEGs closely associated with lactylation status. Intersecting these genes with the single-cell-derived candidates yielded a set of lactylation-related candidate prognostic genes.

Building on the above analyses, univariate Cox proportional hazards regression was performed in the TCGA cohort to systematically screen for prognosis-associated genes significantly linked to OS. The resulting candidate genes were subsequently incorporated into a LASSO Cox regression model to refine and select the most stable risk features, thereby constructing a lactylation-related multigene risk-scoring model. We next evaluated, within the TCGA cohort, the associations of the risk score with OS and clinical characteristics and, together with immune-infiltration analysis, expression of immune checkpoint molecules (e.g., PD-L1, PD-L2), TIDE scores, and predicted drug sensitivity, systematically characterized the immunologic and therapeutic implications of the model. Finally, the predetermined genes and their regression coefficients were transferred to the external two CGGA cohorts to compute the risk score, and the model’s stability and generalizability were validated through survival analyses and assessments of predictive performance.

**Figure 1:**
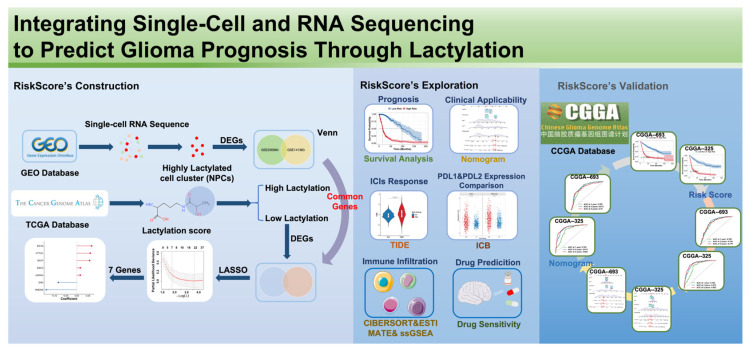
Study flow chart.

The workflow of this study is illustrated in Figure 1.

### 2.2. Lactylation Scoring and Differential Gene Analysis Based on Cell-Type Annotation

**Figure 2:**
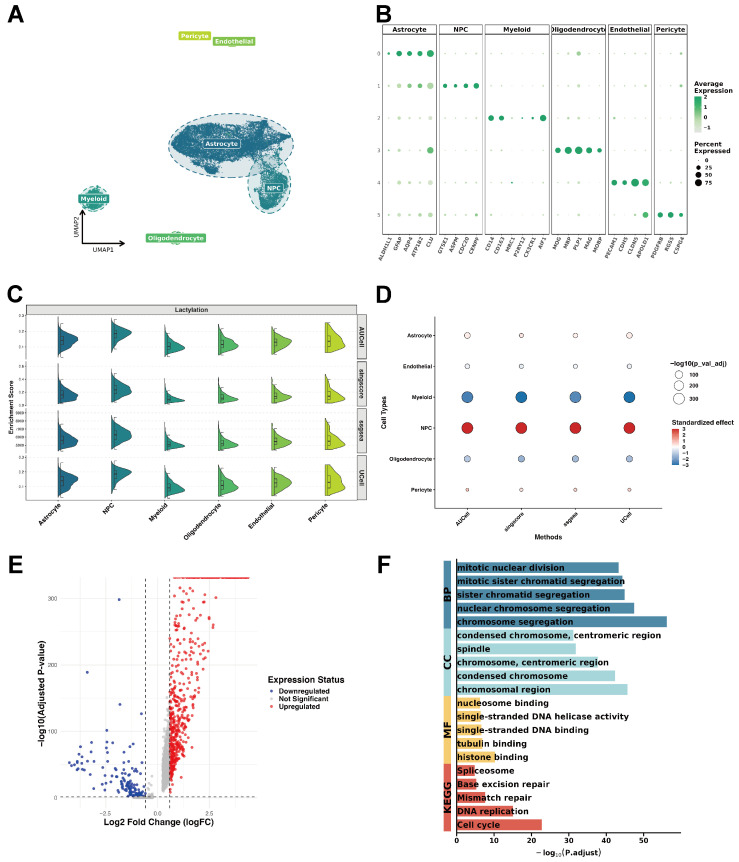
Lactylation-related gene landscape in the GSE141383 scRNA-seq glioma dataset. **(A)** UMAP visualization of major cell populations. (**B**) Dot plot showing expression levels of canonical marker genes across different cell clusters. (**C**) irGSEA-derived expression patterns and distribution of lactylation-related genes across cell types. (**D**) Direction and magnitude of enrichment for lactylation-related signatures across cell types. (**E**) Volcano plot illustrating genes with differential expression between the NPC cluster and all other clusters, with dashed vertical lines indicate |log_2_FC| = 0.585, and the dashed horizontal line indicates the significance threshold of adjusted *p*-value = 0.05 (shown as −log_10_(0.05)). (**F**) GO and KEGG enrichment analyses.

Following quality control, the GSE141383 dataset comprising 23,604 cells and 28,284 genes was subjected to downstream analysis. Cell-type annotation was performed by leveraging both cell markers from the original publication and canonical brain cell markers from the CellMarker 2.0 database. This identified six major cell populations, such as astrocytes, NPCs, myeloid cells, oligodendrocytes, endothelial cells, and pericytes. All types of cells were depicted with UMAP and DotPlot representations (Figure 2A,B). Lactylation-related gene set activity was quantified across all cell types using four scoring algorithms (AUCell, UCell, singscore, and ssGSEA) within the irGSEA. Across all methods, NPCs consistently exhibited the highest lactylation scores, with statistically significant differences compared with other cell types (Figure 2C,D). The results indicate that lactylation might be an important factor in the biological functions of NPCs.

A similar analytical workflow was applied to the GSE200984 single-cell dataset. Seven cell types were ultimately annotated: astrocytes, myeloid cells, neurons, oligodendrocytes, endothelial cells, NPCs, and OPCs (oligodendrocyte precursor cells), followed by dimensionality reduction and visualization (Figure S1A,B). To ensure our findings, the lactylation scoring analysis was repeated in GSE200984, and again observed that the NPC cluster displayed the highest and most significant lactylation scores among all cell types (Figure S1C,D).

Next, DEGs in NPCs relative to all other cell types were identified. Lowly expressed genes were first filtered using logFC.threshold = 0.25 and min.pct = 0.1, followed by more stringent selection based on abs(log_2_FC) > 0.585 and an adj. *p*-value < 0.05. In the GSE141383 dataset, 755 DEGs were identified (Figure 2E). GO and KEGG analyses were then conducted to functionally characterize these DEGs (Figure 2F), revealing significant enrichment in cell-cycle regulation, chromosome segregation, DNA repair, and DNA replication. Applying the same pipeline to the GSE200984 dataset yielded 1411 DEGs, which were likewise enriched in the same pathways (Figure S1E,F). Finally, 406 overlapping genes between the two datasets were identified and defined as NPC-DEGs.

Given that NPCs are characterized by rapid proliferation, we further regressed out S-phase and G2M-phase scores using a linear model in both GSE200984 and GSE141383 datasets. The analysis of residuals revealed two key findings. First, when stratifying NPCs by cell cycle phase, NPCs in the G1 phase (non-proliferative state) still exhibited significantly higher lactylation residuals compared to other non-malignant cell types (Figure S7A,C), suggesting that hyper-lactylation is an intrinsic metabolic feature rather than a transient state during division. Second, to verify consistency across patients, we compared the average lactylation levels of NPCs versus non-NPCs within each individual sample. The results showed that NPCs consistently displayed higher lactylation activity than other cells in nearly every patient (Figure S7B,D), confirming the robustness of this signature.

### 2.3. Lactylation Scoring and Analysis in the TCGA-GBM and TCGA-LGG Cohorts

**Figure 3:**
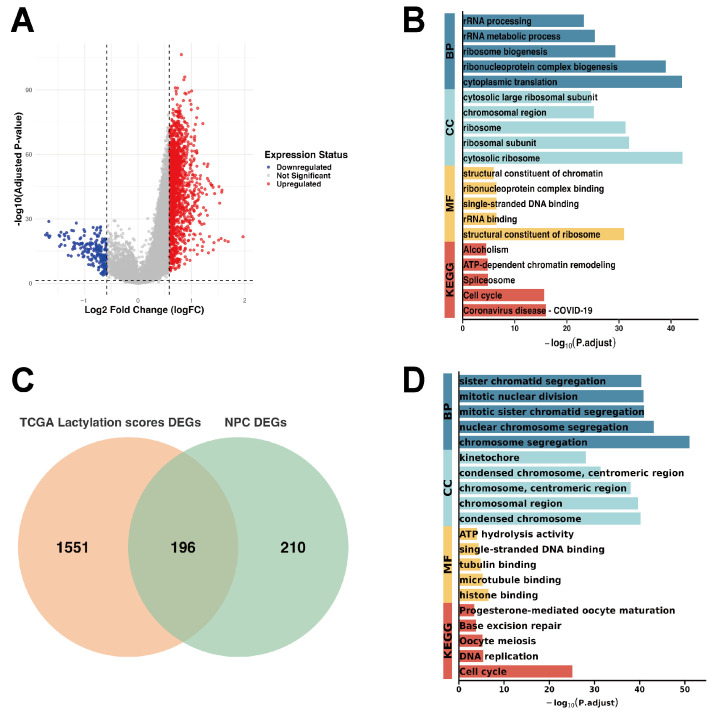
Analysis of lactylation-related genes and DEGs in the TCGA cohort. (**A**) Volcano plot of DEGs between high- and low-lactylation groups, with dashed vertical lines indicate |log_2_FC| = 0.585, and the dashed horizontal line indicates the significance threshold of adjusted *p*-value = 0.05 (shown as −log_10_(0.05)). (**B**) GO and KEGG enrichment of DEGs. (**C**) Venn diagram showing the overlap between lactylation score-associated DEGs in the TCGA cohort and those in NPC. (**D**) GO and KEGG enrichment of overlapping genes.

GSVA-based lactylation scores were independently computed for the TCGA-GBM and TCGA-LGG cohorts. The two scored datasets were subsequently merged, and batch effects were corrected, resulting in a combined cohort of 763 samples. Patients were then classified into high- and low-lactylation groups based on the median lactylation gene score. DEGs between the two groups were identified using the criteria abs(log_2_FC) > 0.585 and an adj. *p*-value < 0.05, yielding a total of 1747 DEGs (Figure 3A). GO and KEGG enrichment analyses (Figure 3B) showed that these genes were predominantly enriched in ribosome biogenesis and increased protein translational activity, accompanied by proliferation-associated biological processes, including cell-cycle regulation and chromatin remodeling.

Intersecting the NPC-DEGs with the lactylation-associated DEGs from the TCGA cohort resulted in 196 overlapping genes (Figure 3C). GO and KEGG enrichment analyses showed that these genes primarily participate in cell division, DNA replication and repair, microtubule organization, and chromatin regulation (Figure 3D). Collectively, these findings underscore the critical roles of the overlapping genes in maintaining genomic stability and promoting cellular proliferation, linking them to tumor initiation, progression, and cell-cycle dysregulation.

Although the top-ranked GO/KEGG terms differed across cohorts, this variation is expected because the differentially expressed gene (DEG) sets were derived from distinct modalities and contrasts (cell-type-specific NPC DEGs from scRNA-seq versus sample-level lactylation-associated DEGs from bulk RNA-seq). The scRNA-seq-derived NPC DEGs primarily reflect cell-intrinsic proliferative programs and genome maintenance processes. In contrast, lactylation-associated DEGs from bulk RNA-seq additionally capture tissue-level biosynthetic activity and shifts in cell-type composition, leading to stronger enrichment of rRNA processing, ribosome biogenesis, and translation-related terms. Notably, convergence emerges when focusing on the intersecting gene set, which consistently implicates chromosome segregation and cell-cycle pathways, including DNA replication and DNA repair, across cohorts.

### 2.4. Development of a Risk Model and a Clinical Nomogram

**Figure 4:**
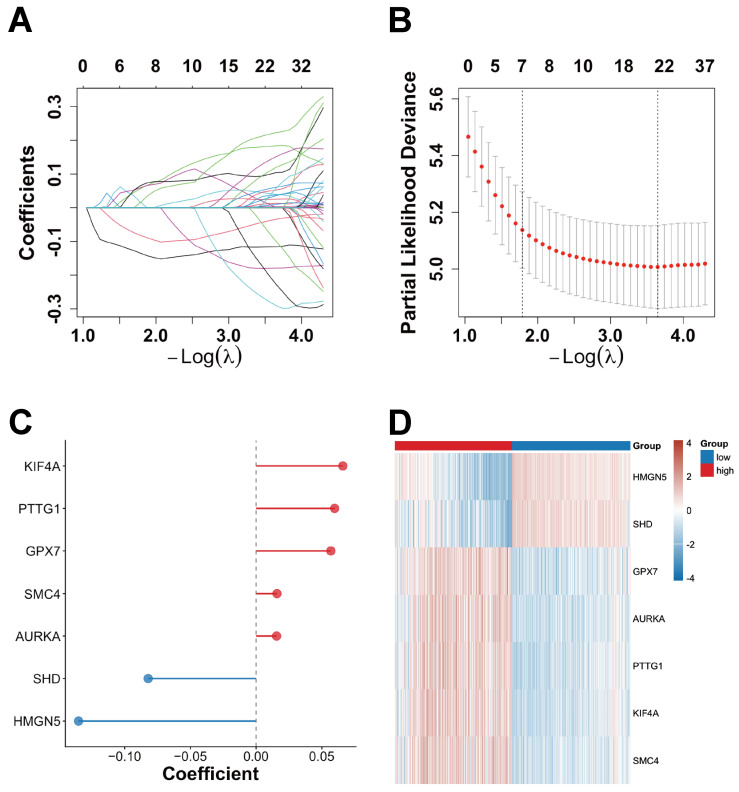
Construction of prognostic gene signatures using LASSO–Cox regression in the TCGA cohort. (**A**) LASSO–Cox coefficient trajectories across −log(λ) with nonzero features (each curve represents one gene). (**B**) Cross-validation curve of partial likelihood deviance, where red dots indicate the mean deviance (±1 SE) and vertical dashed lines denote λmin and λ1se. (**C**) Coefficients for each gene, with the dashed line indicating coefficient = 0 and red/blue points denoting positive/negative coefficients. (**D**) Heatmap displaying the expression of seven genes across risk groups.

Univariate Cox regression analysis identified 169 prognosis-associated genes from the initial set of 196 candidates. Subsequent LASSO regression (Figure 4A,B) refined these to seven core prognostic genes. In this study, the optimal penalty parameter (λ) was selected using the one-standard-error (1-SE) rule. Specifically, within the range defined by the cross-validated minimum deviance ± 1 standard error, we chose the largest λ (λ_1se). Compared with directly adopting λ_min, λ_1se imposes stronger shrinkage on the regression coefficients and, without substantially increasing the cross-validation error, yields a sparser model with fewer variables, a more concise structure, and greater stability across data partitions. This strategy reduces overfitting, enhances interpretability and generalizability, and facilitates variable selection and clinical translation, thereby providing greater practical value for the present study. Genes with nonzero coefficients at λ_1se were defined as the LASSO–Cox prognostic signature genes.

Based on their regression coefficients, these genes were categorized as either positive or negative contributors to risk (Figure 4C). Specifically, KIF4A, PTTG1, GPX7, SMC4, and AURKA were positively associated with higher risk, while SHD and HMGN5 were linked to decreased risk.

Risk scores were computed for all TCGA patients and used to group the cohort into high- and low-risk categories based on the median value. Higher risk scores correlated with elevated expression of risk genes (Figure 4D) and were associated with significantly poorer OS and increased mortality (Figure S2).

**Figure 5:**
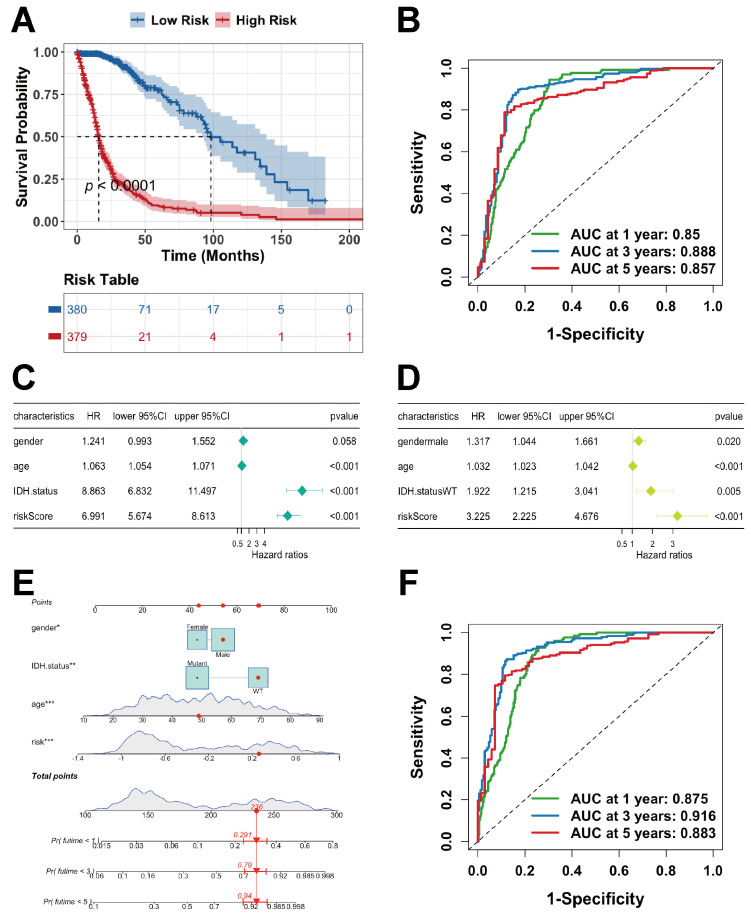
Development of a model and nomogram in the TCGA cohort. (**A**) K-M curves comparing different risk groups. (**B**) ROC curves evaluating the risk score. (**C**) Univariate Cox regression of clinical variables and the risk score. (**D**) Multivariate Cox regression of clinical variables and the risk score. (**E**) Lactylation-related gene-based risk model and nomogram integrating clinical characteristics (significance is indicated by asterisks as defined in Section 4.15). (**F**) ROC curves assessing nomogram performance.

K-M curves demonstrated a significant survival difference between the two groups (*p* < 0.0001), with the high-risk group exhibiting markedly shortened survival (Figure 5A). To assess predictive performance, ROC curves were generated (Figure 5B), yielding AUC values of 0.850, 0.888, and 0.857, respectively, indicating strong predictive performance for survival outcomes.

To further evaluate clinical applicability, univariate and multivariate Cox regression analyses were conducted using age, sex, IDH mutation status, and the risk score (Figure 5C,D). Multivariate analysis confirmed that age, sex, risk score (*p* < 0.001), and IDH mutation status (*p* = 0.005) were independent prognostic factors. A clinical nomogram integrating these variables with the risk score was constructed, showing that lower total points corresponded to higher predicted survival probability (Figure 5E). ROC curve analysis of the nomogram produced AUC values of 0.875 (1 year), 0.916 (3 years), and 0.883 (5 years) (Figure 5F), supporting its strong clinical utility in glioma prognostication.

Risk scores were also calculated independently for the TCGA-GBM and TCGA-LGG datasets. In the GBM cohort, patients stratified by the pre-specified cutoff exhibited notably lower survival in the high-risk group (*p* = 0.038) (Figure S3A). However, predictive performance was modest, with AUC values of 0.589 (1 year), 0.538 (3 years), and 0.508 (5 years) (Figure S3B). This limited performance is likely due to the very small number of low-risk GBM patients (*n* = 2), restricting discriminative power in GBM. It should be noted that the risk score cutoff was pre-specified in the pan-glioma setting and applied unchanged to all validation analyses, including GBM-only subsets. Consequently, when restricting to GBM, the risk-score distribution may be shifted toward higher values, potentially leading to an imbalanced split under the same cutoff.

In contrast, the LGG cohort showed highly significant separation of survival curves, with the low-risk group demonstrating markedly improved outcomes (*p* < 0.0001) (Figure S3E). The model exhibited strong predictive capability in LGG, with AUC values of 0.885 (1 year), 0.857 (3 years), and 0.778 (5 years) (Figure S3F).

Nomograms were further developed for GBM and LGG separately. In the GBM subgroup, age emerged as the principal prognostic determinant (Figure S3C), and the ROC analysis indicated moderate predictive performance, with AUC values of 0.635, 0.721, and 0.691 at 1, 3, and 5 years (Figure S3D). In the LGG cohort, age and risk score were the dominant prognostic indicators (Figure S3G). The predictive accuracy remained high, with AUC values of 0.927, 0.902, and 0.837 (Figure S3H), with only a slight decline over time, indicating that the model provides stable and robust short-term prognostic predictions.

### 2.5. Validation of the Risk Model and the Nomogram

**Figure 6:**
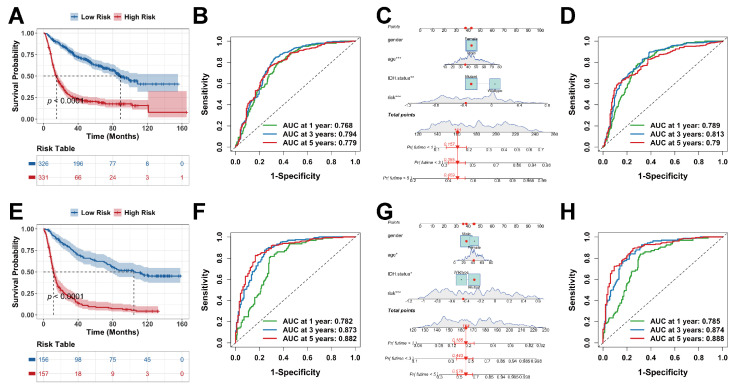
External validation of the model and nomogram in the CGGA-693 (**A**–**D**) and -325 (**E**–**H**) cohorts. (**A**,**E**) K-M curves comparing different risk groups. (**B**,**F**) ROC curves evaluating the risk score. (**C**,**G**) Lactylation-related gene-based risk model and nomogram integrating clinical characteristics (significance is indicated by asterisks as defined in Section 4.15). (**D**,**H**) ROC curves assessing nomogram performance.

The prognostic risk model was further confirmed through testing in two separate external cohorts (CGGA-693 and CGGA-325) using the same analytical procedures described above. In both datasets, the model effectively separated patients into high- and low-risk groups with significantly different survival outcomes (Figure 6A,B,E,F). Evaluation of the clinical nomogram confirmed that age, risk score, and IDH mutation status were consistent and independent prognostic factors across both validation cohorts. Among these, the risk score demonstrated particularly strong predictive power. (Figure 6C,G). In the CGGA-693 cohort, the AUC values were 0.789, 0.813, and 0.790, respectively (Figure 6D). In the CGGA-325 cohort, the corresponding AUCs were 0.785, 0.874, and 0.888 (Figure 6H), collectively supporting the robust predictive capacity of the nomogram.

Within the CGGA-GBM subgroup, the model also demonstrated significant survival discrimination between different risk patients (Figure S4A,E). However, the associated AUC values indicated only moderate predictive performance (Figure S4B,F). Similarly, in the CGGA-693 cohort, the nomogram showed moderate prognostic accuracy (Figure S4C,D). In contrast, in the CGGA-325 GBM cohort, IDH mutation status and the risk score emerged as key factors in predicting outcomes (Figure S4G). The AUC values were 0.606, 0.733, and 0.780, respectively, with a slight increase over time, suggesting improved long-term predictive ability (Figure S4H).

In the CGGA-LGG subgroup, the model likewise provided clear separation between different risk patients (Figure S5A,E), accompanied by excellent predictive performance as reflected in the AUC values (Figure S5B,F). In both LGG-specific nomograms, the risk score consistently emerged as the dominant prognostic variable (Figure S5C,G). The AUC values all exceeded 0.7 (Figure S5D,H), indicating strong prediction accuracy in this subgroup.

Together, these validation results demonstrate that the model exhibits strong discriminative ability and stable performance across independent CGGA datasets of mixed-grade gliomas. Its predictive performance, while somewhat reduced when applied to individual GBM or LGG cohorts—a finding consistent with our observations in the TCGA cohorts and likely attributable to smaller sample sizes—remains robust. Overall, the risk model represents a robust and reliable tool for predicting glioma patient survival and shows substantial promise for individualized risk stratification. Moreover, the nomogram, which integrates lactylation-associated gene signatures with clinical parameters, provides strong clinical prognostic value and may facilitate more personalized decision-making in glioma management.

Next, this study employed a stepwise nested Cox model to assess whether the risk score could provide additional prognostic information beyond established molecular factors. The incremental prognostic value of the risk score was evaluated in the glioblastoma cohort (Table S1). In the CGGA-325 cohort, adding the risk score to standard clinical variables (including IDH status) yielded a highly significant improvement in prognostic accuracy for the whole GBM population (Likelihood Ratio Test *p* < 0.001). Consistent with this, the risk score also provided significant incremental value within the specific IDH-wildtype subgroup in this dataset (*p* < 0.01). In contrast, predictive independence was not statistically significant in the TCGA or CGGA-693 cohorts.

### 2.6. Development of Prognostic Genes

Boxplots were generated to illustrate the expression levels of prognostic genes across various tissue types.

**Figure 7:**
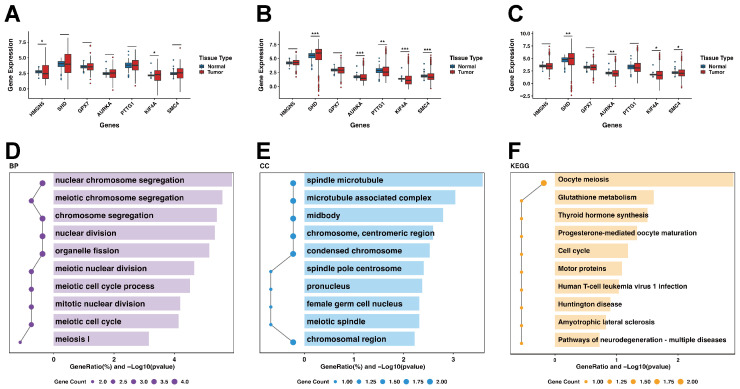
Differential expression and functional enrichment of prognostic feature genes (significance is indicated by asterisks as defined in Section 4.15). (**A**) Expression of seven prognostic genes in normal brain versus GBM. (**B**) Expression of seven prognostic genes in normal brain versus LGG. (**C**) Expression of seven prognostic genes in normal brain versus combined GBM+LGG. (**D**) GO Biological Process enrichment. (**E**) GO Cellular Component enrichment. (**F**) KEGG pathway enrichment.

In the TCGA-GBM dataset, compared to normal brain tissues from GTEx, KIF4A was notably upregulated, whereas HMGN5 was downregulated in tumor tissues (Figure 7A). Comparison of the TCGA-LGG cohort with GTEx revealed that SHD was upregulated, while AURKA, PTTG1, KIF4A, and SMC4 were downregulated in tumor samples (Figure 7B). When the entire TCGA glioma cohort was compared with GTEx, SHD remained consistently upregulated, whereas AURKA, KIF4A, and SMC4 were generally downregulated (Figure 7C).

Overall, SHD exhibited uniform overexpression in glioma tissues, while AURKA, KIF4A, and SMC4 showed a tendency toward reduced expression. Notably, KIF4A was selectively upregulated in GBM, suggesting a potential association with tumor grade or intratumoral cellular heterogeneity.

Functional enrichment analysis of the prognostic gene set identified 66 enriched GO terms (including biological processes and cellular components) and 10 KEGG pathways, with the top enriched terms displayed (Figure 7D–F). Specifically, the length of the bars indicates the significance level ($-{Log}_{10}\;pvalue$), while the position and size of the dots represent the GeneRatio and Gene Count, respectively. Both GO and KEGG results indicated that these genes mainly play important roles in meiosis processes and related regulatory pathways.

**Figure 8:**
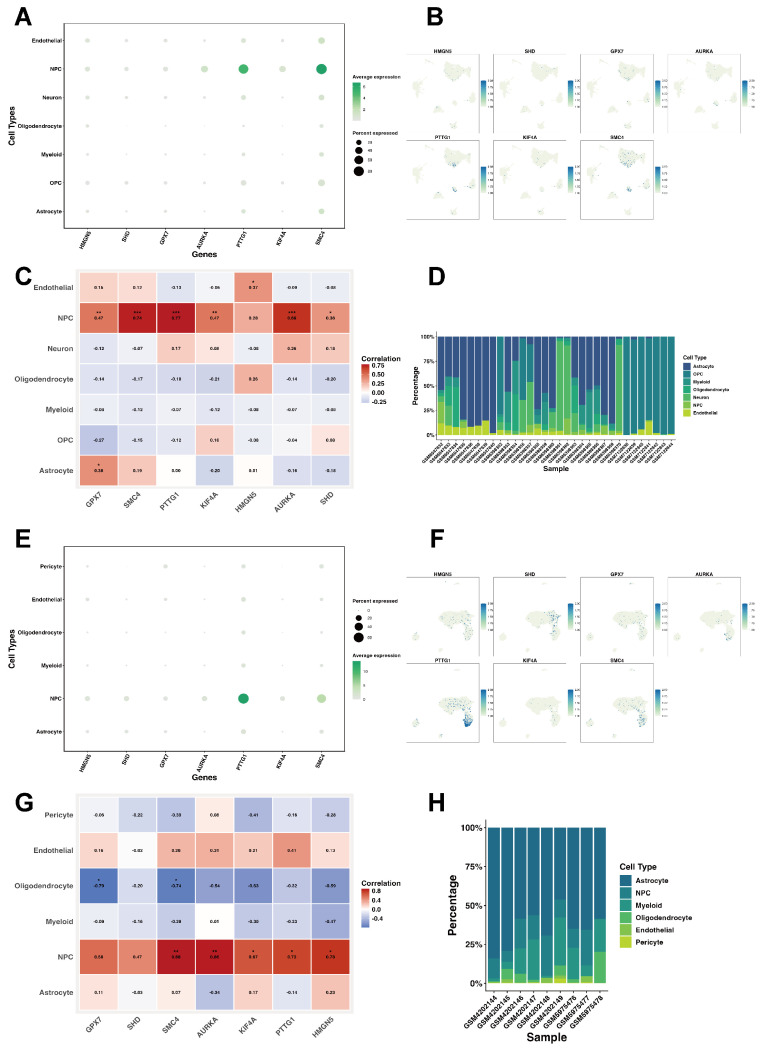
Single-cell expression of prognostic feature genes. (**A**) Dot plot showing gene expression in GSE200984. (**B**) Distribution of the seven prognostic genes in GSE200984. (**C**) Heatmap of Pearson correlations between cell types and gene expression in GSE200984 (significance is indicated by asterisks as defined in Section 4.15). (**D**) Cell-type composition per sample in GSE200984. (**E**) Dot plot showing gene expression in GSE141383. (**F**) Distribution of the seven prognostic genes in GSE141383. (**G**) Heatmap of Pearson correlations between cell types and gene expression in GSE141383 (significance is indicated by asterisks as defined in Section 4.15). (**H**) Cell-type composition per sample in GSE141383.

The expression characteristics of prognostic genes within the GSE200984 dataset were further explored. Distinct expression heterogeneity across cell types was observed. Dot-plot analysis (Figure 8A) showed that most prognostic genes were highly expressed in NPCs, with genes such as PTTG1 and SMC4 exhibiting particularly strong expression signals. UMAPs corroborated these findings (Figure 8B). Within the GSE200984 dataset, the prognostic gene expression showed a positive correlation with the proportion of NPCs, with SMC4, PTTG1, and AURKA showing the strongest associations (Figure 8C). Cell-type composition analysis further demonstrated that NPCs represented a substantial fraction of the cellular population in most samples, though notable inter-sample variability existed (Figure 8D). Repeating the same analyses in the GSE141383 dataset yielded highly consistent results (Figure 8E–H).

### 2.7. Risk Score, Immune Microenvironment, and Immunotherapy

**Figure 9:**
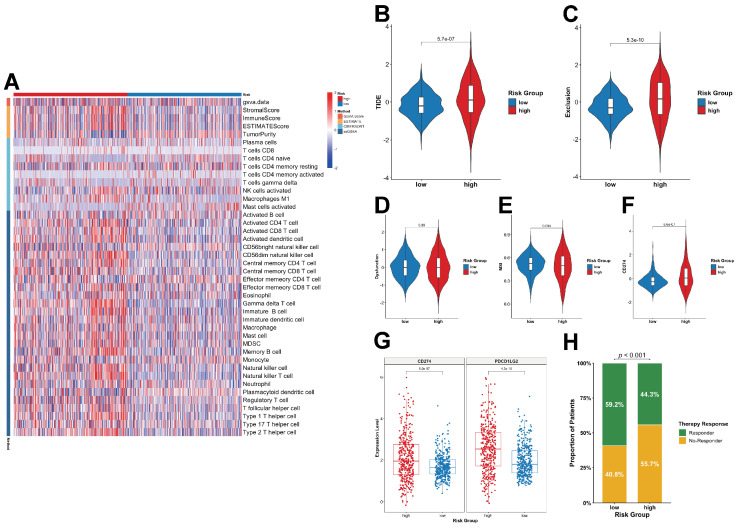
Analysis of immune cell infiltration and immunotherapy responses between risk groups in the TCGA cohort. (**A**) Heatmap of immune cell infiltration. (**B**) TIDE Score. (**C**) Exclusion Score. (**D**) Dysfunction Score. (**E**) MSI Score. (**F**) CD274 Expression. (**G**) Expression of Immune Checkpoint Genes. (**H**) Proportion of Immunotherapy Responders.

To elucidate the biological mechanisms underlying the risk score, first, the characteristics of the TME between different risk strata were compared (Figure 9A). A strong positive correlation existed between the lactylation score and the risk score.

Additionally, the high-risk group displayed increased immune and stromal infiltration but decreased tumor purity, indicating that the lactylation-based risk score is tightly linked to the cellular composition and complexity of the TME. These findings indicate that high-risk patients harbor a more heterogeneous and immunologically active TME.

Given the observed association between the risk score and TME characteristics, the tumor immune escape potential was subsequently evaluated using the TIDE algorithm. High-risk patients exhibited markedly higher TIDE and Exclusion scores (Figure 9B,C), implying enhanced immune evasion capacity and a potentially diminished response to ICI (immune checkpoint inhibitor) therapy. In contrast, median Dysfunction scores were comparable between the two groups and did not differ significantly (Figure 9D). The high-risk group also showed slightly lower MSI levels than the low-risk group, although this difference was not statistically significant (Figure 9E). Assessment of predicted ICI responsiveness indicated that a smaller proportion of high-risk patients were classified as likely responders compared with the low-risk group (Figure 9H), suggesting that low-risk patients may derive greater therapeutic benefit from ICI treatment. Furthermore, expression levels of immune checkpoint-related genes PD-L1 and PD-L2 were elevated in the high-risk group (Figure 9G), consistent with CD274 (PD-L1) expression patterns inferred from the TIDE analysis (Figure 9F).

### 2.8. Risk Score and Drug-Treatment Analysis

**Figure 10:**
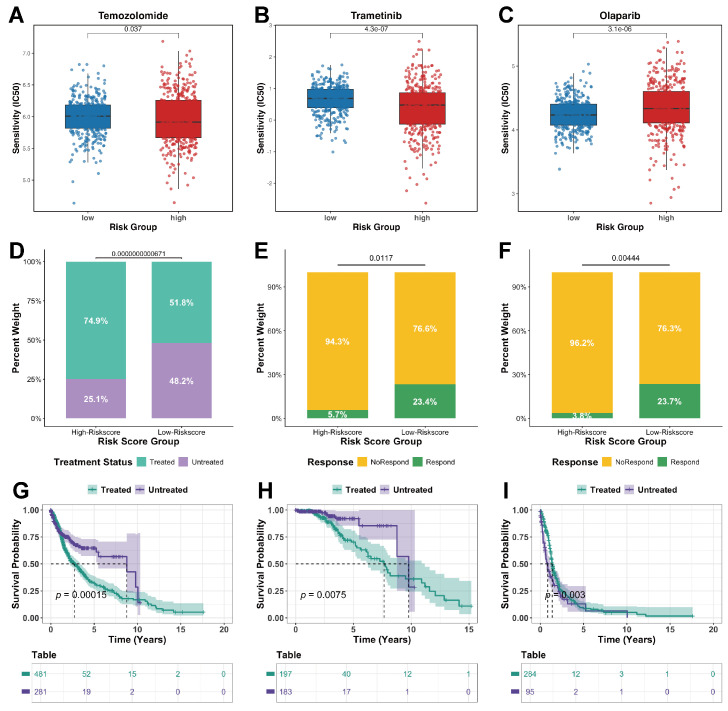
Drug sensitivity, treatment response, and survival by risk score in the TCGA cohort. (**A**) Sensitivity of Temozolomide. (**B**) Sensitivity of Trametinib. (**C**) Sensitivity of Olaparib. (**D**) Differences in treatment status. (**E**) Comparison of response rates to overall drug therapy. (**F**) Distribution of clinical response to chemotherapy. (**G**–**I**) K-M survival curve illustrating survival differences between treated and untreated patients in the three cohorts (overall, low-risk, high-risk).

Analysis of drug sensitivity revealed a distinct response pattern between the risk groups: the high-risk group exhibited lower predicted IC_50_ values (indicating higher sensitivity) for Temozolomide and Trametinib, whereas the low-risk group showed lower predicted IC_50_ values for Olaparib (Figure 10A–C). This differential response highlights the potential of the risk model to guide tailored therapeutic strategies.

In the TCGA cohort, treated and untreated patients were compared. The analysis showed that high-risk patients were more likely to receive pharmacological intervention, consistent with their more severe disease status (Figure 10D). Moreover, the analysis of the distribution of clinical responses to overall drug therapy and chemotherapy revealed that low-risk patients exhibited significantly higher response rates (Figure 10E,F), demonstrating their superior treatment responsiveness.

Kaplan–Meier analyses in the TCGA cohort revealed risk- and time-dependent treatment effects. Untreated patients showed better overall survival in the full cohort and in the low-risk subgroup (Figure 10G,H), an effect mainly driven by significantly improved long-term (≥2 years) outcomes (Figure S6D,E), whereas short-term (<2 years) differences were modest and not significant (Figure S6A,B). In contrast, high-risk patients benefited from therapy, with significantly better overall survival in treated individuals (Figure 10I) and a clear short-term advantage (Figure S6C), while the long-term benefit remained a non-significant trend (Figure S6F).

Overall, low-risk patients exhibit stronger therapeutic responsiveness, yet they are less likely to choose pharmacological intervention due to milder disease severity. Meanwhile, untreated low-risk patients maintain excellent long-term prognosis. In contrast, high-risk patients—who typically present with more aggressive disease—are more likely to be treated, and pharmacological therapy may yield short-term clinical benefits in this group but does not necessarily provide sustained long-term improvement.

## 3. Discussion

For decades, lactate was considered a byproduct of anaerobic glycolysis; however, accumulating evidence indicates that it also contributes to energy metabolism [14], cellular homeostasis [15], and signaling regulation [16,17]. Since its discovery in 2019, lactylation—an emerging PTM—has been recognized as a regulatory mechanism implicated in diverse pathological processes, including cancer [18]. In gliomas, the Warburg effect results in lactate accumulation that provides metabolic fuel and biosynthetic substrates, thereby supporting rapid tumor growth and infiltration into surrounding brain tissue [19]. Thus, lactylation is not simply a metabolic consequence but may actively promote malignant progression and contribute to the poor prognosis of gliomas. Accordingly, developing a prognostic model based on lactylation-related features may help elucidate tumor biology and inform clinical decision-making.

Prognostic models based on lactylation-associated genes have shown significant associations with survival across multiple cancer types. Earlier studies proposed lactylation as a potential prognostic indicator in gastric cancer and melanoma [20,21]. However, the relationship between lactylation-related gene signatures and clinical outcomes remains incompletely defined, underscoring the need for more rigorous investigation.

More recent investigations have begun to address these uncertainties. However, most studies rely on tissue-level (bulk) analyses without single-cell resolution, limiting robust risk stratification across glioma subtypes. Furthermore, they focus primarily on prognostic prediction and provide limited guidance for clinical decision-making, which substantially reduces practical utility.

Single-cell analysis showed that lactylation-related genes are predominantly expressed in NPCs. The enrichment of lactylation signatures in NPCs may reflect a “neural progenitor–like malignant metabolic state.” This pattern differs from differentiated glial cells: astrocytes can export lactate via lactate transport mechanisms, whereas oligodendrocytes couple glycolysis to myelin maintenance [22,23]. In contrast, NPCs rely on the Warburg effect to support biomass synthesis. This configuration may increase intracellular lactate and lactyl–coenzyme A, creating a substrate-rich environment that can promote histone lactylation [24].

By integrating GSVA scores derived from transcriptomic data in the TCGA cohort, seven lactylation-related genes that formed the foundation for developing a lactylation-related risk score model were identified. This model was subsequently externally validated and used to build a nomogram.

Among the seven prognosis-related genes, KIF4A encodes a microtubule-associated motor protein of the kinesin-4 subfamily. KIF4A is required for accurate chromosome organization and segregation during mitosis, thereby supporting genomic stability. KIF4A is frequently overexpressed in lung, breast, colorectal, prostate, and glioma cancers, and higher expression is associated with poorer prognosis [25]. PTTG1 is highly expressed across multiple malignancies and is associated with reduced survival, suggesting potential diagnostic and prognostic value [26]. GPX7, an antioxidant enzyme, is upregulated in several malignancies. In papillary thyroid carcinoma, higher GPX7 expression has been associated with greater tumor burden [27], suggesting that GPX7 may indicate more aggressive disease. SMC4 is also elevated in sarcoma, gastric, breast, and liver cancers; in sarcoma, its expression tends to increase with immune cell infiltration [28]. AURKA, a serine/threonine kinase, is broadly overexpressed across many solid tumors [29,30]. Accumulating evidence suggests that AURKA interacts with the glycolytic enzyme Lactate Dehydrogenase B (LDHB); AURKA-mediated phosphorylation of LDHB may enhance the Warburg effect, indicating a metabolic component of AURKA-driven oncogenic activity [31]. Dysregulated AURKA has also been implicated in hepatic metastasis in colorectal cancer [32]. Succinate dehydrogenase (SDH) is a core enzyme of the tricarboxylic acid (TCA) cycle and is often aberrantly expressed in cancers, including gastrointestinal stromal tumors, pheochromocytomas, and paragangliomas [33]. HMGN5 has recently emerged as a potential epigenetic therapeutic candidate in breast cancer [34].

GO and KEGG enrichment analyses indicate that these genes are enriched in key biological pathways, particularly those related to meiosis and chromosome segregation. This enrichment supports the hypothesis that coordinated dysregulation of these genes may impair genomic stability and thereby promote tumor progression.

Specifically, elevated expression of KIF4A, PTTG1, SMC4, and AURKA in NPCs suggests that lactylation is closely linked to cell-cycle regulation in this proliferative compartment. These four genes are canonical regulators of mitosis. Because lactate accumulation driven by aerobic glycolysis is metabolically coupled to rapid proliferation, their expression may partly capture the proliferative burden of high-risk tumors. In lung adenocarcinoma, the high-risk group was enriched for hypoxia-related pathways, and PTTG1 was proposed to promote angiogenesis and support tumor-cell survival and the vascular niche via HIF-1α (hypoxia-inducible factor 1-alpha) signaling [35]. Consistently, KIF4A has been reported to promote glioblastoma progression by modulating the HIF1A–VEGFA axis [36]. Hypoxia is a major driver of lactate accumulation and lactylation, although lactate can also increase under aerobic glycolysis. Together, these findings suggest a hypoxia–HIF-driven metabolic program that supports lactate production and supplies substrates for lactylation.

In breast cancer, circKIF4A has been reported to promote metastasis by reprogramming glucose metabolism. This suggests that KIF4A participates in a metabolism–proliferation coupling network [37]. SMC4 downregulation may induce a diapause-like state and trigger metabolic reprogramming in tumor cells. It has also been reported to relieve transcriptional constraints on enzymes in the glycolytic “investment phase,” increasing glycolytic flux and lactate accumulation. Elevated lactate may further increase histone lactylation, including H4K12la [11,38]. These observations suggest that SMC4 may function not only in cell division but also upstream of lactylation-related epigenetic regulation. Accordingly, SMC4 may contribute to the model not merely as a proliferation marker but also as an indicator of lactylation-associated metabolic reprogramming.

AURKA activation depends on its cofactor TPX2. TPX2 can be lactylated at K249 (TPX2-K249la), which has been reported to prevent phosphatase PP1 from binding AURKA and thereby sustain AURKA phosphorylation and activation [39]. In addition, AURKA can phosphorylate LDHB, potentially enhancing the Warburg effect and increasing lactate production [31].

Although HMGN5 and SHD were included in the lactylation-related gene signature, they carried negative coefficients in our risk model. This pattern suggests that, unlike other putative risk drivers, HMGN5 and SHD may reflect intrinsic protective programs or a differentiation bias, potentially limiting metabolic reprogramming or maintaining a less invasive phenotype [40,41].

Based on this gene set, we developed a lactylation-related risk score with potential prognostic value in glioma. In the TCGA cohort, high-risk patients had worse survival than low-risk patients, supporting the prognostic performance of the model. ROC analyses further supported its predictive accuracy. These results were replicated in two external validation cohorts (CGGA-693 and CGGA-325), strengthening confidence in model generalizability across independent datasets.

To improve clinical utility, we integrated the risk score with standard clinical variables to construct a nomogram. The resulting nomogram showed stable performance in two CGGA cohorts, supporting its potential applicability across patient populations.

We also observed an association between the risk score and the TME. High-risk tumors exhibited a more complex and heterogeneous immune landscape. TIDE analysis suggested increased immune evasion in high-risk patients, indicating that they may be less responsive to ICIs. However, analyses of ICB response and CD274 (PD-L1) expression suggested that a subset of high-risk patients may still benefit from immunotherapy, underscoring the multifactorial determinants of treatment response.

Drug sensitivity profiling further supported the clinical relevance of the model by revealing distinct therapeutic sensitivities between risk groups. The risk score appeared to correlate with both drug responsiveness and the durability of treatment benefit. In high-risk patients, our results suggest that pharmacotherapy may provide only short-term benefit, largely through transient cytotoxic effects that are insufficient to eradicate rapidly proliferating tumor subsets that drive relapse. In contrast, in low-risk patients, better long-term survival among untreated individuals suggests that the survival gain from pharmacotherapy may be modest, whereas cumulative toxicity could compromise long-term quality of life.

In summary, our lactylation-based risk model may help advance precision oncology for glioma. By integrating scRNA-seq with clinical bulk RNA-seq, we developed and validated a seven-gene prognostic risk score. At the single-cell level, we identified NPCs as the predominant population with high lactylation activity and found that lactylation was associated with immune exclusion and chemoresistance, pointing to potential therapeutic vulnerabilities in glioma. Finally, incorporating the risk score into a clinical nomogram provides a quantitative decision-support tool that links molecular risk with clinical variables and supports individualized treatment planning.

Building on these analyses, we propose a translational framework to convert this bioinformatic advance into clinically actionable benefit. In routine practice, clinicians could record age, sex, and IDH mutation status, quantify expression of the seven core genes (e.g., by PCR or sequencing), and compute an individualized risk score for stratification. The clinical variables and risk score can then be entered into the nomogram. Predictor-specific points are summed to obtain a total score, which is mapped to the survival axis to estimate OS probabilities.

For patients classified as high risk, the model indicates elevated PD-L1 expression yet limited clinical benefit. Future immunotherapy trials may therefore need to move beyond PD-1 monotherapy and prioritize combination strategies aimed at overcoming immune exclusion. Conversely, for low-risk patients, watchful waiting or low-intensity single-agent therapy may be preferable to upfront aggressive chemoradiotherapy, potentially reducing treatment-related toxicity while maintaining disease control.

Nonetheless, several limitations should be acknowledged. Regarding the gene signature, the initial pool of lactylation-related genes was derived from pan-cancer studies and may therefore be biased toward conserved metabolic-stress or proliferation programs. However, such overlap is consistent with the role of lactylation in linking aerobic glycolysis to cell-cycle progression. By intersecting these candidates with NPC-specific markers, we aimed to reduce non-specific stress signals and isolate the component more likely to be co-opted by stem-like glioma cells to promote malignant progression.

In addition, some patient subgroups were small, which may limit statistical power and reduce confidence in subgroup-specific inferences. We used stepwise nested Cox model comparisons to quantify the incremental prognostic information provided by the risk score beyond standard molecular factors. Significant incremental value was observed in CGGA-325, including the IDH-wildtype GBM subgroup; however, this improvement was not consistently significant in TCGA or CGGA-693. This variability may reflect differences in cohort composition and limited power in specific subsets. Further validation in larger, well-annotated multicenter GBM datasets will be important to clarify generalizability and clinical applicability and to define the clinical contexts in which the risk score provides consistent incremental benefit.

## 4. Materials and Methods

### 4.1. Data Sources

The TCGAbiolinks package (version 2.36.0) was used to download the TCGA-GBM and TCGA-LGG datasets from the TCGA database (http://portal.gdc.cancer.gov; accessed on 12 July 2025) [42]. The TCGA-GBM dataset included 260 patients (259 with survival data), and the TCGA-LGG dataset included 505 patients (504 with survival data). Additionally, the CGGA-GBM and CGGA-LGG datasets were obtained from the China Glioma Genome Atlas (CGGA) database (http://www.cgga.org.cn; accessed on 1 October 2025), specifically from the CGGA-693 and CGGA-325 cohorts. The histological diagnoses in these retrospective cohorts were based on the metadata provided by the databases, which align with the 2016 World Health Organization (WHO) Classification of Tumors of the Central Nervous System [43]. Although the 2021 WHO classification has introduced updates such as the use of Arabic numerals and distinct molecular markers for grading [6], we retained the 2016 terminology to preserve the statistical integrity of these historical datasets. In this study, consistent with the TCGA and CGGA cohort definitions, GBM corresponds to WHO grade IV, while LGG comprises WHO grades II and III [44]. Consequently, in the CGGA datasets, patients were screened as follows: 249 WHO grade IV cases in CGGA-693 GBM and 139 in CGGA-325 GBM; 443 WHO grade II-III cases in CGGA-693 LGG and 182 in CGGA-325 LGG. Single-cell RNA sequencing (scRNA-seq) datasets GSE200984 and GSE141383 were retrieved from the Gene Expression Omnibus (GEO) database (http://www.ncbi.nlm.nih.gov/geo; accessed on 28 July 2025). GSE200984 comprises samples from eight primary gliomas and eight recurrent GBMs [45], while GSE141383 includes cells derived from surgical specimens of 66 glioma patients [46]. Furthermore, 270 normal cerebral cortex tissue samples were obtained from the Genotype-Tissue Expression (GTEx) project (http://commonfund.nih.gov/GTEx; accessed on 16 July 2025). A total of 322 lactylation-related genes were compiled from previous studies [47,48,49].

### 4.2. ScRNA-Seq Analysis

Quality control and analysis used the Seurat package (version 5.3.0) [50]. Genes expressed in fewer than three cells were excluded, along with low-quality cells expressing fewer than 300 genes. The proportions of mitochondrial, ribosomal, and hemoglobin genes were calculated, retaining only cells with <15% mitochondrial content, >3% ribosomal content, and <0.1% hemoglobin content. MALAT1, mitochondrial genes, ribosomal genes, and putative doublets were excluded from downstream analyses. Data were normalized with NormalizeData and scaled with ScaleData. Highly variable genes (*n* = 2000) were identified using FindVariableFeatures, and RunPCA was applied to conduct principal component analysis (PCA). Batch effects were adjusted with the Harmony function. Clustering used the first 20 principal components with FindClusters (resolution = 0.1).

Cell-type identification was performed using marker genes reported in previous studies and those curated from the CellMarker 2.0 database (http://bio-bigdata.hrbmu.edu.cn/CellMarker/; accessed on 28 August 2025) [45,46]. The annotated cell populations were visualized using the uniform manifold approximation and projection (UMAP) and DotPlot, providing an intuitive overview of the cellular landscape and classification.

### 4.3. Lactylation Gene Set Enrichment Scoring in Cell Clusters

Lactylation gene set enrichment scores were computed using irGSEA with AUCell, UCell, singscore, and ssGSEA [51]. Score distributions across clusters were visualized using half-violin plots. Results across methods were integrated using the irGSEA integrate function by combining significance levels.

Next, the FindAllMarkers function was used to identify differentially expressed genes (DEGs) in the neural progenitor cell (NPC) cluster, applying thresholds of abs(log_2_FC) > 0.585 and an adj. *p*-value < 0.05. DEGs shared by both single-cell datasets were defined as NPC-DEGs.

To separate lactylation activity from proliferation effects, we performed cell-cycle regression. Cell-cycle scores (S.Score and G2M.Score) were computed per cell using Seurat’s CellCycleScoring. Subsequently, a linear regression model was constructed:

(1) $$Y=\beta_{0}+\beta_{1}S+\beta_{2}G2M+\epsilon$$

where Y represents the robust Z-transformed lactylation score. We then extracted the residuals (ϵ) from this model. These residuals represent the “corrected” lactylation activity that cannot be explained by cell cycle status alone.

For each sample, we calculated the mean residual score in NPCs and non-NPCs and used their difference to quantify patient-level enrichment.

### 4.4. Analysis of Lactylation Gene Activity

Lactylation scores were computed in TCGA-GBM and TCGA-LGG using Gene Set Variation Analysis (GSVA) (version 2.2.0) [52]. The two datasets were merged, and batch effects were corrected by the removeBatchEffect function from the limma package (version 3.64.3) [53]. Patients were stratified into high- and low-lactylation groups using the median score. DEG analysis between the two groups was conducted using the limma package [53], applying thresholds of abs(log_2_FC) > 0.585 and an adj. *p*-value < 0.05 to identify DEGs. These DEGs represent molecular features associated with distinct levels of lactylation activity.

Candidate genes were defined as the overlap between bulk DEGs and NPC-DEGs using VennDiagram (version 1.7.3) [54].

### 4.5. Enrichment Analysis of Candidate Genes

Functional enrichment was performed using clusterProfiler (version 4.16.0) [55]. The Gene Ontology (GO) and the Kyoto Encyclopedia of Genes and Genomes (KEGG) analyses were performed to explore the biological functions and pathways linked to the candidate genes. Pathways and terms with both *p*-values and *q*-values < 0.05 were considered statistically significant.

### 4.6. Construction of a Lactylation-Related Risk Score Model

To develop a prognostic model associated with lactylation, the Least Absolute Shrinkage and Selection Operator (LASSO) regression was conducted utilizing the glmnet package to distinguish prognosis-related genes [56]. A risk score model can be built based on the chosen genes by:

(2) $$\mathrm{Risk}\;\mathrm{Score}\;=\;\sum_{j=1}^{n}Coef(Gene)\times Expr(Gene)$$

where Coef (Gene) denotes the LASSO regression coefficient of each gene and Expr (Gene) represents the corresponding gene expression level.

### 4.7. Model Validation and Evaluation of Predictive Performance

Patients were grouped into high- and low-risk categories according to their median risk score. Kaplan–Meier (K-M) curves, using the survminer and survival packages [57], compared OS between groups. Time-dependent receiver operating characteristic (ROC) curves at 1, 3, and 5 years were generated using timeROC [58]. The area under the curve (AUC) values were calculated to quantify discrimination and the dashed diagonal line represents the performance of a random classifier (AUC = 0.5).

### 4.8. Construction of the Nomogram

To identify independent prognostic factors and evaluate the clinical relevance of the risk score model, univariate and multivariate Cox regression analyses were performed, incorporating variables such as age, sex, IDH mutation status, and the calculated risk score. A nomogram was later created with the rms package to forecast OS probabilities [59]. The nomogram’s ability to predict outcomes was assessed employing ROC curves created with the timeROC package (version 0.4) [58], and AUC values were calculated with the dashed diagonal line represents the performance of a random classifier (AUC = 0.5).

### 4.9. Assessment of Incremental Prognostic Value

To quantify the additional prognostic information provided by the risk score beyond established clinical factors, we performed a stepwise multivariate Cox regression analysis. Baseline models were constructed using standard clinical variables (Age, Gender) and available molecular features (IDH mutation status, MGMT promoter methylation, and 1p/19q codeletion). The risk score was then added to these baseline models to create combined models. Incremental value was assessed using Harrell’s C-index, Akaike information criterion (AIC), and likelihood ratio test (LRT) [60]. Lower AIC indicates improved fit with parsimony.

### 4.10. Analysis of Prognostic Signature Genes

Expression data from the TCGA-GBM and TCGA-LGG cohorts were combined with normal cerebral cortex tissue data from the GTEx database. Batch effects were rectified utilizing the remove BatchEffect function from the limma package [53]. The Wilcoxon rank-sum test compared prognostic gene expression between tumor and normal tissues, and boxplots were generated to visualize differential expression patterns.

Functional enrichment of prognostic genes was performed using clusterProfiler (GO/KEGG) [55]. Terms with *p* < 0.05 and *q* < 0.05 were considered significant.

To examine the cellular distribution of prognostic genes, the DotPlot function in Seurat was used to calculate average expression levels across different cell types. The table function was used to count the absolute number of each cell type per sample, followed by normalization using the prop.table function to derive cell-type proportions. Finally, correlations between cell-type proportions and prognostic gene expression were assessed.

### 4.11. Immune Microenvironment Analysis and Prediction of Immunotherapy Response

Relative fractions of 22 immune cell types were inferred using CIBERSORT [61]. To further characterize the tumor microenvironment (TME) in relation to the risk score, the ESTIMATE algorithm was employed to compute the Stromal Score, Immune Score, ESTIMATE Score, and Tumor Purity [62]. Additionally, the enrichment levels of various immune cell populations were assessed using the ssGSEA function within the GSVA package [52]. The results were visualized as a heatmap using pheatmap (version 1.0.13) [63].

To predict potential responses to immunotherapy, the Tumor Immune Dysfunction and Exclusion (TIDE) algorithm (http://tide.dfci.harvard.edu/; accessed on 6 October 2025) was employed [64]. Higher TIDE scores indicate greater immune evasion and a lower likelihood of response to immune checkpoint inhibitors (ICIs) [64]. Responder data based on the TIDE score predicted responder status. The Exclusion Score quantified the degree of T-cell exclusion from the TME and reflected the T-cell impairment level [65]. The microsatellite instability (MSI) Score indicated the expression of the MSI gene set [66]. CD274 (PD-L1) expression was analyzed as a biomarker related to the immune checkpoint blockade (ICB) response [67].

### 4.12. ICB Analysis

Based on ICB-related genes (PD-L1 and PD-L2) [68], the Wilcoxon rank-sum test was applied to evaluate expression differences between the different risk groups.

### 4.13. Drug Sensitivity Analysis

With the oncoPredict package (version 1.2) [69], drug sensitivity and the prediction of potential therapeutic agents for patients categorized as high- or low-risk were assessed. Drug response data were obtained from the Genomics of Drug Sensitivity in Cancer (GDSC) database (https://www.cancerrxgene.org; accessed on 4 October 2025), specifically from the GDSC2 dataset.

Using get.oncoPredict.res, ridge regression models were trained to predict half-maximal inhibitory concentration (IC_50_) values in TCGA-GBM/LGG samples. Afterwards, a Wilcoxon rank-sum test was used to compare the predicted IC_50_ values between two risk groups, where lower IC_50_ values suggest higher drug sensitivity.

### 4.14. Evaluation of Drug Treatment Response and Survival Benefit Across Risk Subgroups

Clinical supplementary information was downloaded using the TCGAbiolinks package [42], and drug-treatment-related clinical data were extracted. First, the proportions of different risk patients who received systemic pharmacological treatment were compared. Treatment response was redefined in accordance with the clinical outcome annotations: Complete response and Partial response were categorized as Respond, whereas Clinical progressive disease and Stable disease were classified as NoRespond. Patients with missing or ambiguous response information were excluded from subsequent analyses.

Then, separate analyses for overall drug therapy and chemotherapy were conducted. Chi-square tests compared Respond vs. NoRespond proportions between risk groups, and stacked bar plots summarized response distributions.

Follow-up and survival data were merged with treatment annotations across GBM and LGG cohorts. Based on these combined data, patients were grouped by treatment received or not. Survival analyses were subsequently performed in the overall cohort and within the high- and low-risk strata. Kaplan–Meier curves were generated, with stratified analyses for short-term (OS < 2 years) and long-term (OS > 2 years) survival. A *p*-value < 0.05 was deemed statistically significant.

### 4.15. Statistical Analysis

To compare the continuous variables between the two groups, the Wilcoxon rank-sum test was used to examine the relationships between categorical variables, and the chi-square test was employed. A *p*-value of less than 0.05 was considered statistically significant. Specifically, significance was indicated as ns (*p* ≥ 0.05), * (*p* < 0.05), ** (*p* < 0.01), and *** (*p* < 0.001). All R packages used are open-source and free. All statistical analyses were carried out using R software (version 4.5.1).

## 5. Conclusions

By integrating two independent glioma scRNA-seq datasets with large-scale bulk transcriptomes, we delineated a cell-resolved lactylation landscape and identified an NPC-like malignant compartment with consistently high lactylation activity. This enrichment persisted after regression of S- and G2M-phase signals, supporting lactylation as a stable metabolic state rather than a surrogate for proliferation. Integrating NPC-enriched genes with highly active lactate-related genes from bulk cohorts, we identified a parsimonious seven-gene lactylation signature (KIF4A, PTTG1, GPX7, SMC4, AURKA, SHD, and HMGN5) and a corresponding risk score that stratified overall survival in TCGA and generalized to two external CGGA cohorts. Incorporating the risk score with key clinical variables yielded a nomogram for individualized survival estimation, improving the clinical translatability of this lactylation-centered framework.

Beyond prognostication, the risk score links lactylation biology to the tumor microenvironment and therapeutic context. High-risk tumors showed increased stromal and immune infiltration and reduced tumor purity, accompanied by higher TIDE and exclusion scores and increased PD-L1/PD-L2 expression, consistent with an immune-evasion phenotype dominated by immune exclusion. Computational drug-response prediction suggested risk-stratified vulnerabilities, with higher predicted sensitivity to temozolomide and trametinib in the high-risk group and to olaparib in the low-risk group. Treatment-annotation analyses further indicated risk- and time-dependent differences in survival benefit with pharmacological intervention. Collectively, this work establishes a lactylation-centered, cell-informed stratification framework that integrates malignant state, immune context, and therapeutic sensitivity, offering a practical biomarker platform for precision risk assessment and trial enrichment in glioma.

## Data Availability

All data used in this study were obtained from the TCGA, GEO, CGGA, and GTEx databases. Detailed information about these datasets can be accessed through their respective official websites. The full names of the databases and their corresponding URLs are provided in the main text or Supplementary Materials.

## Supplementary Materials

The following supporting information can be downloaded at: https://www.mdpi.com/article/10.3390/ijms27041649/s1.
