# Supplementary material for: Integrating Single-Cell and RNA Sequencing to Predict Glioma Prognosis Through Lactylation
Source: Int J Mol Sci. 2026 Feb 8;27(4):1649. doi: 10.3390/ijms27041649 (PMC12940179; doi:10.3390/ijms27041649)
Supplement: Supplementary file 1 [file ijms-27-01649-s001.zip › ijms-4047603-supplementary.pdf]

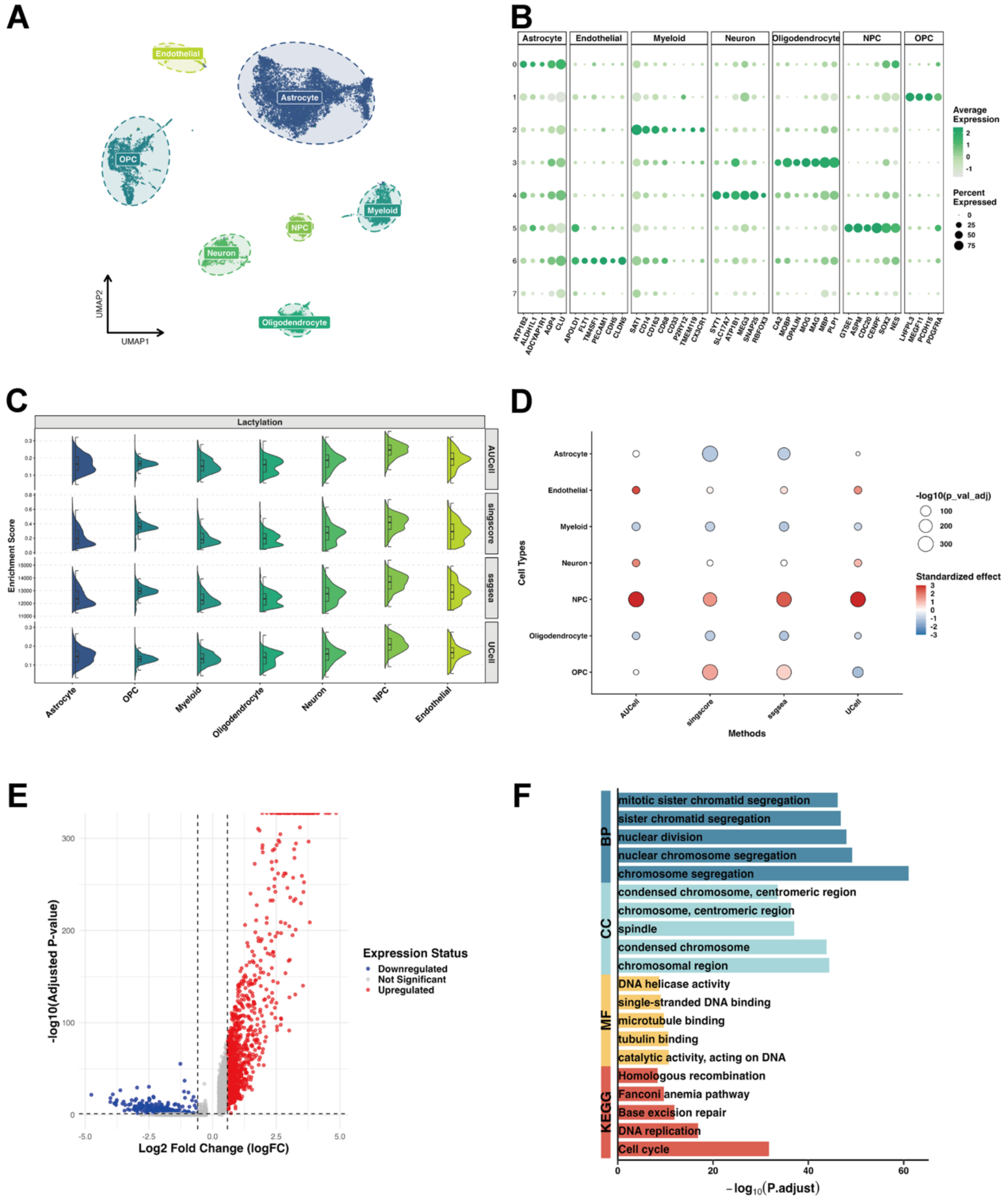

Figure S1. Lactylation-related gene landscape in the GSE200984 single-cell RNA sequencing glioma dataset. **(A)** UMAP visualization of major cell populations. **(B)** Dot plot showing expression levels of canonical marker genes across different cell clusters. **(C)** irGSEA-derived expression patterns and distribution of lactylation-related genes across cell types. **(D)** Direction and magnitude of enrichment for lactylation-related signatures across cell types. **(E)** Volcano plot illustrating genes with differential expression between the NPC cluster and all other clusters, with dashed vertical lines indicate $|\log_2 FC| = 0.585$, and the dashed horizontal line indicates the significance threshold of adjusted p-value = 0.05 (shown as $-\log_{10}(0.05)$). **(F)** GO and KEGG enrichment analyses.

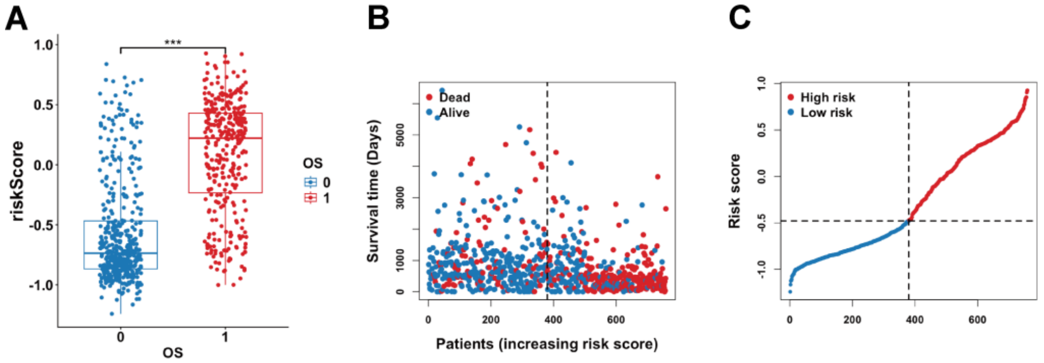

Figure S2. Survival differences between high- and low-risk patients stratified by risk scores. **(A)** Boxplot comparison of risk score differences between surviving and deceased patients (significance is indicated by asterisks as defined in Section 4.15). **(B)** Distribution of patients' survival status and time ordered by increasing risk scores. **(C)**Continuous distribution of patients ordered by increasing risk scores.

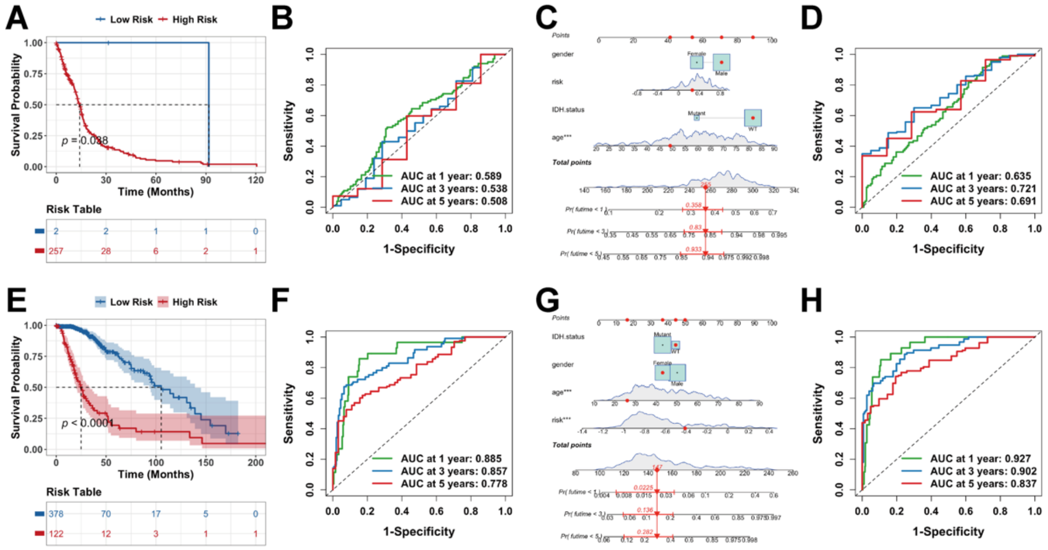

Figure S3. External validation of the risk score and nomogram in the TCGA-GBM and TCGA-LGG cohorts. **(A, E)** Kaplan–Meier survival curves comparing high- and low-risk groups. **(B, F)** ROC curves evaluating the risk score. **(C, G)** Lactylation-related gene–based risk model and nomogram integrating clinical characteristics. **(D, H)** ROC curves assessing nomogram performance.

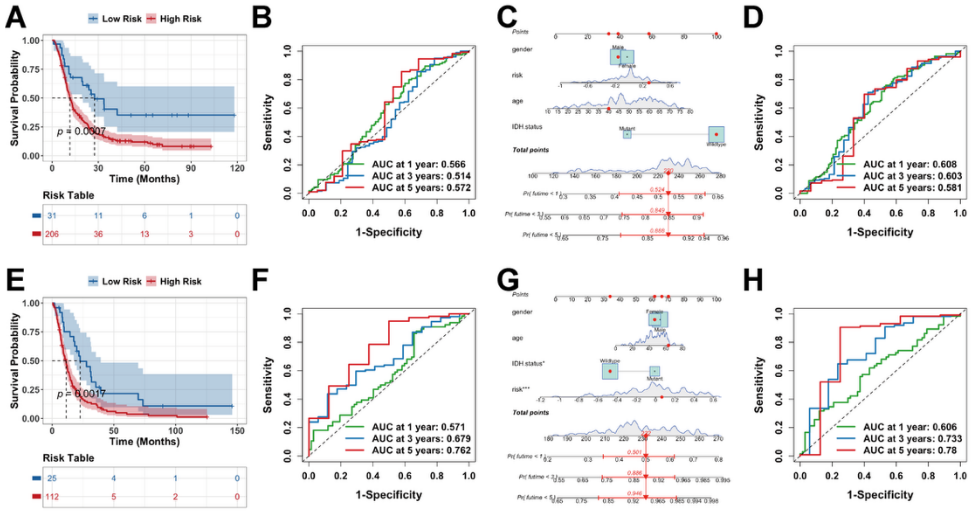

Figure S4. Validation of risk score and nomogram in different CGGA-GBM cohorts. **(A, E)** Kaplan–Meier survival curves comparing high- and low-risk groups. (in 693,325 cohorts). **(B, F)** ROC curves evaluating the risk score (in 693,325 cohorts). **(C, G)** Lactylation-related gene–based risk model and nomogram integrating clinical characteristics (in 693,325 cohorts). **(D, H)** ROC curves assessing nomogram performance (in 693,325 cohorts).

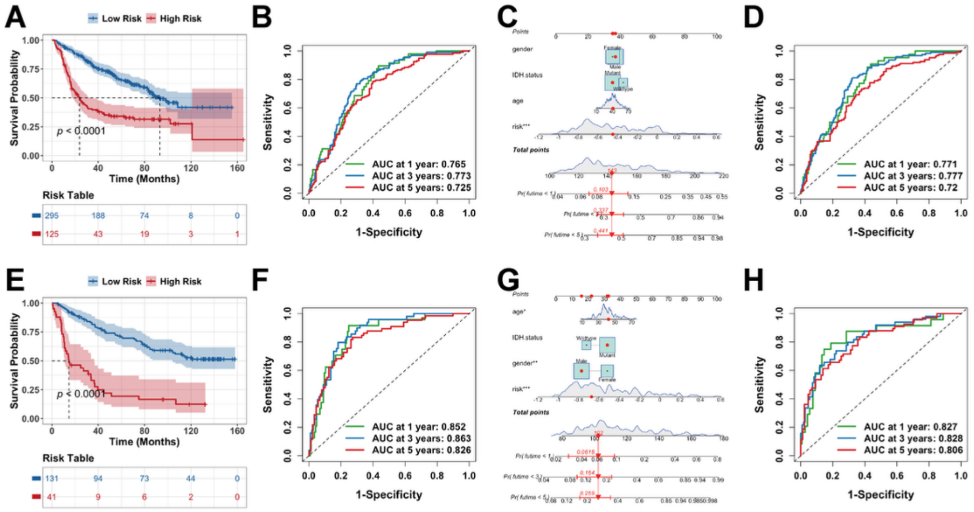

Figure S5. Validation of risk score and nomogram in different CGGA-LGG cohorts. **(A, E)** Kaplan–Meier survival curves comparing high- and low-risk groups. (in 693,325 cohorts). **(B, F)** ROC curves evaluating the risk score (in 693,325 cohorts). **(C, G)** Lactylation-related gene–based risk model and nomogram integrating clinical characteristics (in 693,325 cohorts). **(D, H)** ROC curves assessing nomogram performance (in 693,325 cohorts).

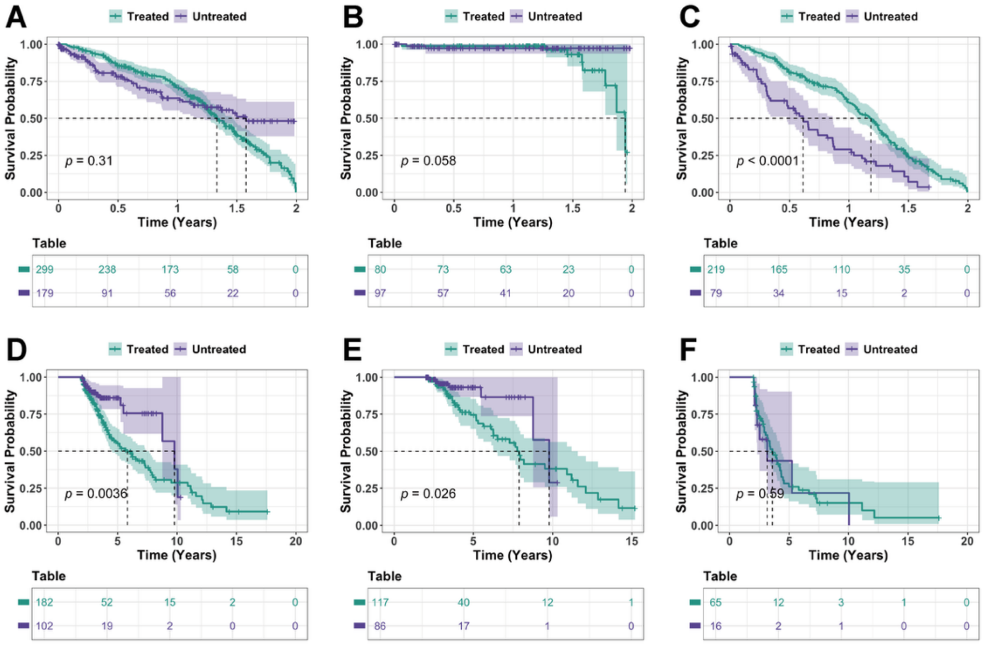

Figure S6. Analysis of short-term (< 2 years) and long-term (≥ 2 years) survival between treated and untreated patients in the TCGA cohort. **(A-C)** Kaplan–Meier survival curve illustrating short-term survival differences in the three cohorts (overall, low-risk, high-risk). **(D-F)** Kaplan–Meier survival curve illustrating long-term survival differences among the three cohorts (overall, low-risk, high-risk).

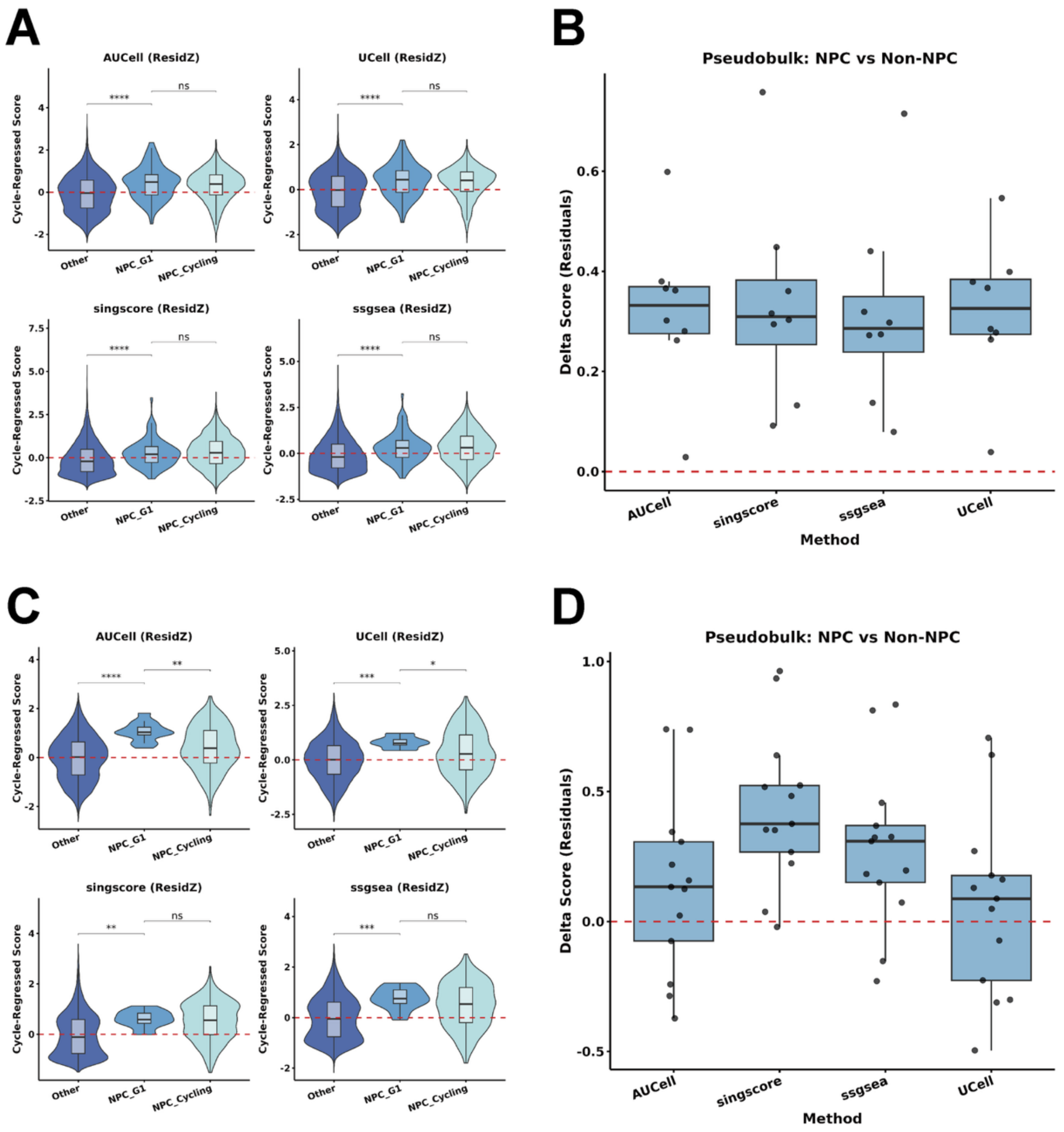

Figure S7. Validation of cell-cycle-independent and robust lactylation enrichment in NPCs (significance is indicated by asterisks as defined in Section 4.15). **(A)** Cell-cycle-regressed lactylation scores (GSE141383). **(B)** Pseudobulk NPC vs non-NPC lactylation delta (GSE141383). **(C)** Cell-cycle-regressed lactylation scores (GSE200984). **(D)** Pseudobulk NPC vs non-NPC lactylation delta (GSE200984).

Table S1. Summary of incremental prognostic value analysis in GBM patients.

Dataset	Subgroup	Baseline Model Variables	Baseline C-index	Combined C-index	ΔC-index	LRT P-value	AIC Comparison
CGGA-325	IDH-wt GBM	Age + Gender	0.545	0.576	+0.031	0.006 (***)	647.9→642.3 (↓)
		+ MGMT	0.534	0.578	+0.044	0.007 (***)	631.3→626.1 (↓)
		+ MGMT + 1p/19q	0.535	0.579	+0.045	0.006 (***)	624.2→618.8 (↓)
	Whole GBM	Age + Gender + IDH	0.520	0.604	+0.084	<0.001 (***)	1003.5→990.7 (↓)
		+ MGMT	0.513	0.607	+0.095	<0.001 (***)	985.9→972.6 (↓)
		+ MGMT + 1p/19q	0.530	0.603	+0.073	<0.001 (***)	958.6→947.2 (↓)
CGGA-693	IDH-wt GBM	Age + Gender	0.525	0.525	0.000	0.996 (ns)	1409.4→1411.4 (↑)
		+ MGMT	0.526	0.529	+0.003	0.893 (ns)	1120.1→1122.1 (↑)
		+ MGMT + 1p/19q	0.542	0.542	+0.001	0.807 (ns)	960.8→962.8 (↑)
	Whole GBM	Age + Gender + IDH	0.567	0.578	+0.011	0.281 (ns)	1784.7→1785.5 (↑)
		+ MGMT	0.568	0.577	+0.009	0.178	1407.1→1407.3

						(ns)	(↑)
		+ MGMT + 1p/19q	0.585	0.584	-0.001	0.182 (ns)	1224.7→1224.9 (↑)
TCGA	IDH-wt GBM	Age + Gender	0.588	0.599	+0.011	0.262 (ns)	1636.9→1637.7 (↑)
		+ MGMT	0.599	0.609	+0.010	0.265 (ns)	1140.1→1140.9 (↑)
	Whole GBM	Age + Gender + IDH	0.624	0.633	+0.009	0.134 (ns)	1739.1→1738.8 (↓)
		+ MGMT	0.643	0.653	+0.010	0.150 (ns)	1217.4→1217.4 (~)

Note: The "Combined Model" adds the Risk Score to the Baseline Model. ΔC-index= Combined C-index-Baseline C-index. LRT = Likelihood Ratio Test.
